# Massive transfusion practices and low‐titer group O whole blood utilization before and after implementation in a low‐volume level I trauma center: A six‐year review

**DOI:** 10.1111/trf.70409

**Published:** 2026-09-26

**Authors:** Chinelo P. Onyenekwu, Debra M. Lehman, Brittany Trower, Paula Woywod, Joseph P. Connor

**Affiliations:** ^1^ Department of Pathology and Laboratory Medicine, Transfusion Medicine Division University of Wisconsin–Madison Madison Wisconsin USA; ^2^ Transfusion Services Laboratory University of Wisconsin Hospital and Clinics Madison Wisconsin USA; ^3^ Department of Nursing ‐ Practice Innovation University of Wisconsin Hospital and Clinics Madison Wisconsin USA

**Keywords:** low‐volume trauma center, massive transfusion, prehospital transfusion, transfusion stewardship, trauma resuscitation, whole blood

## Abstract

**Background:**

Low‐titer O whole blood (LTOWB) has been increasingly incorporated into trauma resuscitation and massive transfusion protocols (MTPs). Recent randomized trials have not demonstrated superiority of prehospital LTOWB over component‐based resuscitation, while the utilization and sustainability of LTOWB programs, particularly in low‐volume trauma systems, remain incompletely characterized.

**Study Design and Methods:**

This was a 6‐year review (April 2019–April 2025) of trauma MTPs at a level 1 trauma center with a low MTP frequency. Transfusion service logs, trauma flowsheets, prehospital and electronic medical records were reviewed for product usage, LTOWB utilization and discard patterns, and clinical outcomes.

**Results:**

One hundred and twenty‐five MTPs were identified (122 component‐only and 11 LTOWB‐supported), with predominantly male (70%) and 81% blunt trauma. Component MTPs had a median RBC utilization of 6 units; 45% required ≤1 MTP line. Prehospital LTOWB use was limited: 33% of units were transfused, and only 2 of 13 patients requiring an MTP received prehospital LTOWB. LTOWB‐supported MTPs typically issued 10 units, and most patients received 1–2 units. Of 524 LTOWB units procured during the first 6 months, 201 were transfused and 323 discarded (62%), with a peak discard rate of 81% early in rollout. Overall, 30‐day mortality was 54%, and 25% occurred within 4 h.

**Conclusions:**

LTOWB use was limited, and early wastage was substantial. LTOWB implementation in low‐volume centers should align inventory and protocols with clinical demand; stewardship measures may reduce waste and improve sustainability.

**Abbreviations**
CPD: citrate‐phosphate‐dextrose
CPDA: citrate‐phosphate‐dextrose‐adenine
ED: emergency department
EMR: electronic medical records
EMS: emergency medical services
IRB: institutional review board
LTOWB: low‐titer group O whole blood
MTP: massive transfusion protocol
RBC: red blood cells
SWiFT: study of whole blood in frontline trauma
TOWAR: type O whole blood and assessment of AGE during prehospital resuscitation

## 1 INTRODUCTION

Massive transfusion protocols (MTPs) are essential in trauma care, aiming to rapidly replace blood volume losses and improve survival. Low‐titer group O whole blood (LTOWB) has regained prominence in trauma resuscitation following evidence from military and civilian settings demonstrating improved outcomes and logistical simplicity and ability to provide red blood cells, plasma, and platelets in a single product.^1^, ^2^, ^3^, ^4^, ^5^, ^6^ However, recent randomized controlled trials, including SWiFT and TOWAR, have not demonstrated superiority of prehospital LTOWB over component‐based resuscitation.^7^, ^8^ Much of the experience with LTOWB implementation and its logistical advantages has originated from high‐volume trauma systems,^9^, ^10^ centers with high rates of penetrating trauma, or military environments with distinct injury patterns and system structures.^2^, ^6^, ^11^, ^12^

Low‐volume trauma centers face fundamentally different operational realities. Massive transfusion protocol activations may be infrequent, mechanisms of injury differ, and blood product turnover often limits LTOWB feasibility.^10^, ^13^ High discard rates, uncertain indications, and evolving protocols further complicate implementation.^14^, ^15^, ^16^ Experience from diverse trauma settings, particularly those with lower MTP frequency, is needed to inform LTOWB utilization, stewardship, and inventory strategies across different operational environments.^6^, ^17^, ^18^

In October 2024, our level 1 trauma center implemented a LTOWB program with integration across prehospital, emergency department (ED), and MTP workflows. The program was developed through multidisciplinary collaboration among trauma surgery, ED personnel, and air medical emergency team members, including clinicians with prior training and experience in a high‐volume LTOWB program. Drawing on this expertise, our initial protocol was modeled on practices from that high‐volume setting and adapted to our local trauma system. Early observations revealed unexpectedly high discard rates, raising questions about optimal stocking, usage patterns, and alignment with clinical need. This study reviewed 6 years of trauma MTPs and the first 6 months of LTOWB implementation, characterizing utilization patterns, wastage, clinical outcomes, and iterative program modifications aimed at improving stewardship and sustainability.

## 2 MATERIALS AND METHODS

### 2.1 Study setting and population

This retrospective study was conducted at a 614‐bed academic level 1 adult trauma center and included all trauma patients who triggered an institutional MTP activation between April 1, 2019, and April 30, 2025. The annual trauma caseload averages 3500. The study was deemed exempt from institutional review board (IRB) review by the University of Wisconsin‐Madison IRB. Patient consent was not required for the study. Inclusion criteria comprised adult trauma patients (≥18 years) with suspected or confirmed hemorrhagic shock who met the trauma service's predefined MTP activation criteria: systolic blood pressure <90 mmHg, heart rate >120 bpm, or evidence of ongoing exsanguination. Pediatric cases and non‐trauma‐related MTP activations were excluded.

Low‐titer O whole blood was introduced into institutional protocols in mid‐October 2024 and sourced from the regional blood supplier as low‐titer O‐positive and O‐negative whole‐blood units. The titer threshold defined by the blood supplier is <200. The product was stored in a monitored blood refrigerator in the ED and the trauma resuscitation bay, with a combination of remote temperature monitoring and daily temperature logs reviewed each day. Prehospital LTOWB availability was limited to designated air medical units trained in transfusion handling and documentation. Key components included stocking 12 units of LTOWB for air ambulance use on a 2‐week rotation and maintaining a dedicated ED refrigerator stock of 4 units. Each LTOWB MTP was designed to allow up to 10 units of LTOWB (two 5‐unit lines) before switching to component therapy (red blood cells (RBC)), plasma, platelets, and cryoprecipitate), aligning with emerging national guidelines for hybrid resuscitation. A patient could receive up to 2 units of LTOWB in the prehospital phase, and 2 units of LTOWB from the ED refrigerator. The component MTP comprised 5 units of RBC, 5 units of plasma in each line, 1 apheresis platelet for every other line, and 2 pooled units of cryoprecipitate added in the third line of therapy for an adult.

### 2.2 Data sources and definitions

Data was obtained from transfusion service MTP logs, trauma flowsheets, ED documentation, prehospital air ambulance transfusion logs, and electronic medical records (EMR). These sources provided data on demographics, mechanisms of injury, interventions, transfusion timing, product use, and mortality for all patients reviewed. Data extraction was conducted by two independent reviewers using a standardized data abstraction form to ensure consistency, with discrepancies reconciled by consensus. Data validation involved cross‐checking between the transfusion service database, trauma registry, and the hospital's EMR system.

Wastage was defined as any WB unit discarded due to expiration or compromised temperature control, expressed as a percentage of total units issued during the same period. Monthly inventory utilization reports were generated to assess temporal trends and identify operational bottlenecks.

LTOWB‐supported resuscitation refers to trauma resuscitation that includes the use of LTOWB at any phase, including prehospital transfusion, transfusion from the ED refrigerator, or MTP.

Early transitioning refers to a patient being transitioned from the issued WB MTP to a component‐only MTP without using up the 2 lines of LTOWB MTP units.

Post‐transfusion MTP refers to transfusions outside of MTP that occurred within 24 h of the start of MTP, and after 24 h of MTP start in patients who were alive.

### 2.3 Outcomes

The primary outcomes studied included LTOWB utilization rate, discard rate, prehospital versus ED refrigerator usage, and MTP transfusion intensity. The secondary outcomes were post‐MTP transfusion needs and 30‐day mortality.

### 2.4 Data analyses

Data was abstracted and analyzed using Microsoft Excel version 2508 (Microsoft 365; Microsoft Corporation) and summarized using descriptive statistics. Continuous variables are presented as medians, ranges, or interquartile ranges, and categorical variables are reported as counts and percentages. Due to the small number of patients receiving LTOWB‐supported resuscitation and the substantial imbalance between groups, formal comparative statistical analyses between LTOWB and component‐only cohorts were not performed. Outcomes are therefore presented descriptively.

## 3 RESULTS

### 3.1 Patient characteristics

Over the 6‐year study period, the average annual trauma case load was 3500. One hundred and twenty‐five trauma MTP activations (0.6%) were identified. The median patient age was 40 years (IQR 26–63), and 87 (70%) patients were male. Most injuries were blunt (101, 81%), predominantly vehicle collision‐related (87, 86%). Prehospital transfusion occurred in 49 (39%) patients. More than half (69, 55%) underwent operative or interventional radiology procedures, and nearly half (60, 48%) required ICU admission (Table 1).

**TABLE 1:** Demographics and early clinical course of patients who received trauma MTPs.

Variable	Total (*n* = 125)	LTOWB‐supported resuscitation (*n* = 12)	Component only (*n* = 113)
Age, year, median [IQR]	40 (26–63)	29 (20–75)	43 (29–63)
Male, *n* (%)	87 (70.0%)	8 (66.7%)	79 (69.9%)
Mechanism of injury			
Blunt, *n* (%)	101 (80.8%)	11 (91.7%)	90 (79.6%)
Penetrating, *n* (%)	24 (19.2%)	1 (8.3%)	23 (20.4%)
ED GCS, median [IQR]	4 (3–14)	5 (3–15)	4 (3–14)
ED to OR and/or IR, *n* (%)	69 (55.2%)	6 (50.0%)	63 (55.8%)
ICU stay, *n* (%)	60 (48.0%)	6 (50.0%)	54 (47.8%)
Prehospital transfusion, *n* (%)	49 (39.2%)	5 (41.7%)	44 (38.9%)

Abbreviations: ED, emergency department; GCS, Glasgow Coma Scale; ICU, intensive care unit; IR, interventional radiology; LTOWB, low titer group O whole blood; OR, operating room.

### 3.2 Component MTP patterns

Component‐only therapy was used in 122 (97.6%) MTP activations during the study period. The median RBC transfusion was 6 units; 55 (45%) required 1 or fewer MTP lines, and 21 (17%) required ≥3 lines.

### 3.3 Prehospital whole blood utilization

During the first 6 months, 12 LTOWB units were stocked in the air ambulance every 2 weeks, and 51 (33%) of these units were transfused. Only 2 of 13 trauma patients who later required MTPs received prehospital LTOWB.

### 3.4 Hospital LTOWB utilization

Among 11 LTOWB MTPs activated from mid‐October 2024 to April 30, 2025, 10 (91%) accessed the ED refrigerator LTOWB before MTP confirmation. Ten units (2 lines) were issued for the MTPs; most patients received 1–2 units (Figure 1). Of patients receiving LTOWB MTP, 4 (36%) transitioned early to component‐therapy MTPs.

**FIGURE 1:**
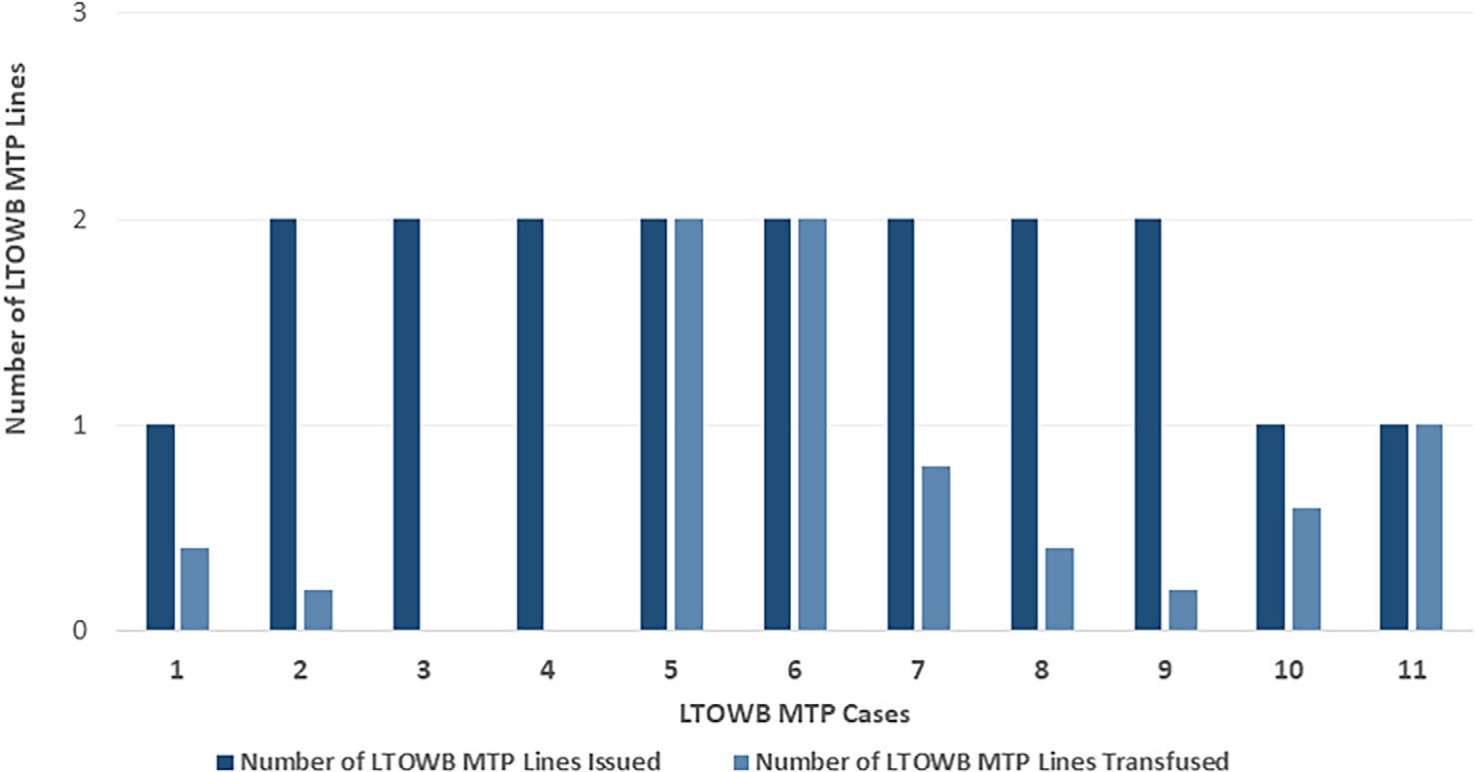
LTOWB MTP lines issued and transfused during LTOWB‐supported trauma resuscitation. For each LTOWB‐supported MTP activation, paired bars represent the number of LTOWB MTP lines issued and the number subsequently transfused. LTOWB, low‐titer group O whole blood; MTP, massive transfusion protocol.

### 3.5 Post‐MTP transfusion and mortality (component‐only, LTOWB incorporated, entire group)

Post‐MTP transfusion within 24 h occurred in 30 of 125 (24%) patients, while 33 of 76 (43%) patients who survived beyond 24 h from MTP start received additional transfusion.

In the whole group, the 30‐day mortality was 54% (68), with 25% (31) occurring within the first 4 h (Table 2). Mortality among patients who received component‐only therapy was 57% (64/113), whereas among patients who received LTOWB‐supported resuscitation, the mortality was 33% (4/12).

**TABLE 2:** Post‐MTP transfusion requirements and 30‐day mortality following LTOWB‐supported or component‐only resuscitation.

Variable	Total (*n* = 125)	LTOWB‐supported resuscitation (*n* = 12)	Component‐only resuscitation (*n* = 113)
Median LTOWB units transfused (range)			
Overall		4 (2–12)	
ED fridge		2 (1–2)^a^	
MTP		2 (0–10)^b^	
Prehospital		3 (2–4)^c^	
Post‐MTP transfusion, *n* (%)	63 (50.4%)	6 (50.0%)	57 (50.4%)
<24 h	30 (47.6%)	2 (33.3%)	28 (49.1%)
≥24 h	33 (52.4%)	4 (66.7%)	29 (50.9%)
30‐day mortality, *n* (%)^d^	67 (53.6%)	4 (33.3%)	63 (55.8%)
<4 h	31 (46.2%)	4 (100.0%)	27 (42.8%)
4–24 h	18 (26.9%)	–	18 (28.6%)
>24 h–29 days	18 (26.9%)	–	18 (28.6%)

*Note*: Due to the small number of patients receiving LTOWB‐supported resuscitation and substantial imbalance between cohort sizes, outcomes are presented descriptively and no formal comparative statistical analyses were performed.

Abbreviations: LTOWB, low‐titer group O whole blood; MTP, massive transfusion protocol.

^a^ For a total of 10 patients with ED fridge LTOWB transfusion.

^b^ For 11 patients who underwent LTOWB MTP.

^c^ For three patients who received LTOWB during prehospital transfusion.

^d^ Percentages for mortality timing are calculated relative to deaths within each subgroup.

### 3.6 LTOWB wastage and protocol refining actions

A total of 524 WB units were procured between October 2024 and April 2025. Of these, 201 units were transfused, and 323 were discarded (62% discard rate). Early discard rates peaked at 81% in the first 2 months of implementation, then declined to 45% following protocol adjustments (Figure 2). A few key protocol adjustments and clinical team communications were made. In late November 2024, we adjusted our standing orders for LTOWB‐ leading to a reduction in air emergency services supply and hospital LTOWB stock. In January 2025, we extended the LTOWB shelf life to 21 days, up from the 14‐day shelf life our service had implemented. In March 2025, we expanded LTOWB use beyond trauma resuscitation to include other services, with a willingness to incorporate it into patient care, including as a supplemental product in some liver transplant surgeries and for gastrointestinal bleeding in the ED. This change involved extensive communication with multiple non‐trauma clinical teams and renaming our designated ‘Trauma MTP’ to ‘Whole Blood MTP’ to signify its broader use in clinical scenarios beyond trauma resuscitation.

**FIGURE 2:**
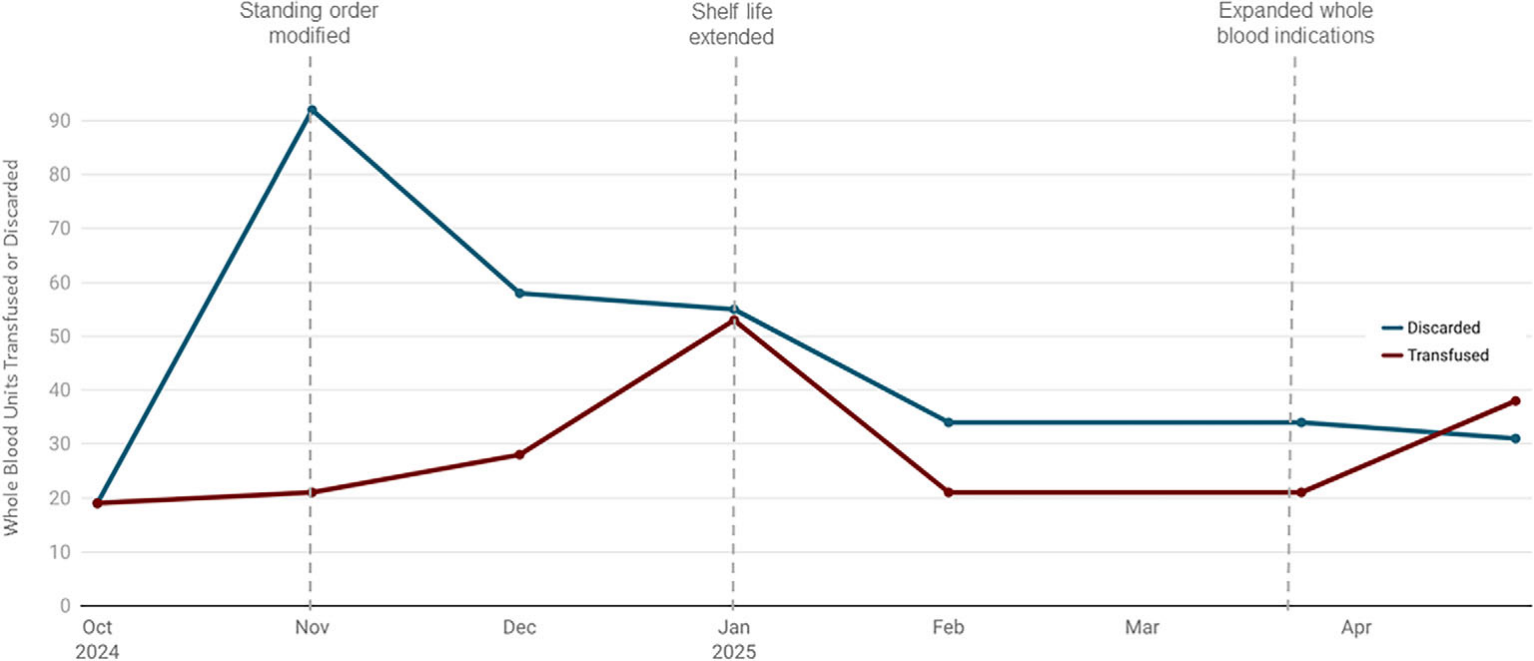
Monthly LTOWB transfusion and discard trends during the first 6 months following program implementation. Dashed vertical lines indicate key operational modifications, including revision of standing order practices, extension of LTOWB shelf life from 14 to 21 days, and expansion of LTOWB utilization beyond trauma resuscitation. LTOWB, low‐titer group O whole blood.

## 4 DISCUSSION

This study reviews trauma MTPs and LTOWB implementation in a low‐volume level 1 trauma center, and focuses on utilization patterns, wastage, and operational performance. The program was initially structured using an inventory model more consistent with higher‐volume LTOWB programs, despite the comparatively low and unpredictable transfusion volume at our center. In addition, because of concerns regarding preservation of platelet function with prolonged storage, LTOWB shelf life was initially limited to 14 days. Retrospective review of MTP activations, utilization, and discard patterns subsequently demonstrated that this initial approach was poorly matched to actual clinical demand and informed iterative modifications to inventory and utilization practices. Several important findings emerge. First, most trauma MTP activations required modest transfusion support, with a median RBC use of 6 units and nearly half requiring one or fewer component MTP lines. Second, LTOWB utilization was limited across prehospital and in‐hospital settings, although ED refrigerator use occurred in most patients requiring a trauma MTP. Component therapy appeared sufficient for a third of trauma cases with early transitioning from LTOWB MTP to component therapy MTP. Third, LTOWB was successfully integrated into trauma resuscitation. However, the process was associated with substantial wastage at the early implementation phase, with a discard rate of 62% during the first 6 months and peaks approaching 81% in the earliest period. Wastages decreased with multiple protocol changes during the first 6 months. Lastly, although observed mortality was 33% among patients receiving LTOWB‐supported resuscitation compared with 57% among those receiving component‐only therapy, the groups were small and unbalanced, and no adjustment was performed for injury severity, mechanism, or other confounders. These findings are therefore descriptive and should not be interpreted as evidence of a treatment effect.

The study findings confirm that while LTOWB implementation in a low‐volume trauma center is feasible, it entails challenges, including limited utilization and high early wastage. This operational reality matches broader trends described in the literature. The practical use of LTOWB in low‐incident settings is constrained by unpredictable trauma cases and rapid product expiration. The initial 62% wastage rate reflected a mismatch between the initial inventory strategy, restricted shelf life, and actual clinical demand. This highlights the importance of early utilization review and iterative program adjustment. The subsequent decline in both LTOWB transfusion and discard after January 2025 occurred in the setting of unpredictable clinical demand and extension of product shelf life, with corresponding adjustments in inventory and restocking practices. This altered product turnover and the timing of utilization and expiration. The initial high waste mirrors the experiences reported by Barmparas et al.,^14^ who reported 42% expiry prior to use, and Phan‐Tang et al., who noted 54% of their LTOWB required reprocessing to RBCs, and 4% went to waste.^15^ Consistent with these findings, optimizing LTOWB supply requires iterative adjustments to inventory levels and close coordination among trauma, emergency medical services, and transfusion services.

The findings on mortality in the studied cohort should be interpreted in light of evolving research on LTOWB. Earlier observational studies and meta‐analyses suggested potential survival benefits with LTOWB, although these studies were susceptible to patient selection and other sources of confounding.^3^, ^4^, ^11^, ^19^, ^20^, ^21^ More recently, the SWiFT and TOWAR trials did not demonstrate a survival advantage of prehospital LTOWB over component‐based resuscitation.^7^, ^8^ In the absence of demonstrated superiority over component‐based prehospital resuscitation, decisions regarding LTOWB implementation and sustainability should consider clinical outcomes alongside stewardship, cost, utilization, and operational feasibility. These considerations may be particularly important in lower‐volume trauma systems, where limited product turnover can substantially affect program sustainability.

Implementation‐focused studies provide additional insight into the practical challenges of LTOWB adoption. Bäckström et al. demonstrated that minimizing wastage requires careful alignment of inventory strategy, utilization patterns, and system‐level workflows.^22^ Similarly, prehospital and in‐hospital implementation studies have shown that while LTOWB programs are feasible, utilization depends heavily on system design, activation criteria, and patient mix.^14^, ^23^, ^24^ In contrast, our findings reflect the early phase of implementation in a lower‐volume trauma system, where limited utilization and high discard rates indicate a mismatch between product availability and actual clinical demand.

Economic sustainability is a major concern for low‐volume centers. Multiple studies have identified wastage and acquisition costs as primary cost drivers, though higher‐volume centers have demonstrated significant cost savings with LTOWB adoption.^25^, ^26^ Strategies to minimize wastage include in silico inventory optimization,^27^ rotation of unused prehospital units to in‐hospital use, and remanufacturing expiring LTOWB into RBC units.^22^ Remanufacturing was explored as a potential strategy at our institution; however, our blood supplier did not offer this service. Because our transfusion service lacked the centrifugation equipment required for in‐house processing, the additional equipment and operational requirements were considered prohibitive for the relatively low volume of units potentially recovered. Small centers must balance maintaining adequate product availability with the inevitability of some waste due to unpredictable trauma volume, though protocol‐based conversion pathways have achieved zero wastage even in Level III centers.^13^ Additionally, prehospital LTOWB deployment, despite its promise, remains underutilized—fewer than 1% of US ground emergency medical services (EMS) agencies have implemented prehospital transfusion programs—due to scope‐of‐practice limitations, regulatory requirements, cost, and logistical obstacles.^28^ Future integration with EMS‐based decision tools, such as the EMS‐G Score or the Prehospital Transfusion Score, may improve the efficiency of real‐time activation and reduce unnecessary product deployment.^29^, ^30^

From a clinical governance perspective, stewardship is essential. Published protocols for LTOWB inventory management in low‐volume centers remain limited; however, several strategies have emerged from institutional experiences, including those from the current review, that directly address wastage reduction. First, avoid overstocking and maintain inventory levels based on actual usage patterns rather than theoretical needs.^13^, ^15^ Second, where operationally feasible, consider reprocessing protocols—some civilian trauma centers convert unused units to red blood cells (RBCs) after 1–2 weeks of storage. High‐volume centers can also absorb short‐dated units from low‐volume facilities.^15^, ^22^ Third, maximize shelf life by utilizing LTOWB for its full regulatory duration (21 days for CPD, 35 days for CPDA‐1) rather than applying earlier cutoffs, though hemostatic function—particularly platelet activity—degrades significantly after 7–14 days regardless of anticoagulant.^31^, ^32^ Fourth, rotation systems provide another avenue to manage waste. For example, Swedish centers rotate unused prehospital LTOWBs for in‐hospital use in cases of major bleeding, achieving wastage rates of 0%–13% across most centers.^22^ EMS facilities can transfer units nearing expiration to high‐transfusion‐volume hospitals where they are more likely to be used.^33^ At our institution, efforts to reduce discard also included expanding LTOWB utilization beyond trauma to selected high‐blood utilization surgical and non‐surgical settings. Transfusion service physicians met with physician leadership from liver transplantation,  cardiothoracic surgery/anesthesia, and emergency medicine (for ED resuscitation of gastrointestinal hemorrhage) to discuss potential use of short dated LTOWB. An agreed‐upon pathway allowed units approaching the initial 14‐day expiration to be used between days 14 and 21 in selected patients when RBCs and plasma units are ordered together and would otherwise have been administered together. This provided an additional opportunity for utilization while preserving LTOWB availability for trauma earlier in its shelf life. Utilization rates and clinical outcomes within these non‐trauma populations were not specifically evaluated in the present study. This dual‐use strategy may prove especially effective when trauma volume is unpredictable, as in smaller centers.

Implementation frameworks should include scheduled audits, periodic discard analyses, and clear transition criteria from LTOWB to components.^6^, ^34^ Ongoing monitoring of utilization and discard rates enables iterative refinement.^35^ A hybrid MTP approach—reserving LTOWB for clearly defined high‐risk cases (severe hemorrhage, high ISS, penetrating trauma, or MTP activation) while continuing component therapy for others—may be the most cost‐effective and sustainable strategy in similar institutions.^6^, ^11^ Institutions should align LTOWB inventory with historical MTP volume and transfusion intensity,^15^, ^27^ target use for patients at the highest risk of severe hemorrhage,^6^ and integrate prehospital and hospital workflows to optimize utilization.^36^, ^37^ The present study shows that adaptive inventory management, effective team communication, and evidence‐based feedback loops can reduce waste without jeopardizing readiness.

Limitations of this study include its single‐center retrospective design, modest sample size, and limited power for outcome comparisons. Temporal changes in trauma patterns and provider practices, along with variations in injury severity and transfusion timing, may also have influenced the observed outcomes. Study strengths include comprehensive 6‐year longitudinal data capturing all trauma‐related MTPs and detailed product‐level tracking. This analysis offers insight into the implementation challenges of LTOWB programs in low‐volume settings. The experience contributes to a growing body of evidence supporting contextualized transfusion strategies. As described in Scandinavian implementation models, low‐volume trauma centers can serve as essential nodes within broader transfusion networks by integrating prehospital and hospital workflows, rotating stock between facilities, and remanufacturing expiring units.^17^, ^22^, ^33^ By sharing utilization data and standardizing triage algorithms,^5^, ^35^ these centers can refine their roles within national LTOWB programs while avoiding the overextension of limited blood resources.

In conclusion, LTOWB implementation in this low‐volume Level 1 trauma center was feasible but initially associated with substantial wastage, which improved following protocol refinement. These findings stress the need to tailor LTOWB programs to institutional trauma demographics and promote data‐driven stewardship to support sustainability. The sustainability of LTOWB programs depends on operational alignment between supply, demand, and clinical practice.

## CONFLICT OF INTEREST STATEMENT

The authors have disclosed no conflicts of interest.

## Data Availability

The data that support the findings of this study are available from the corresponding author upon reasonable request.
